# Acidosis and Phosphate Directly Reduce Myosin’s Force-Generating Capacity Through Distinct Molecular Mechanisms

**DOI:** 10.3389/fphys.2018.00862

**Published:** 2018-07-10

**Authors:** Mike Woodward, Edward P. Debold

**Affiliations:** Muscle Biophysics Lab, Department of Kinesiology, University of Massachusetts, Amherst, MA, United States

**Keywords:** muscle fibers and skeletal, myosins, phosphates, fatigue, acidosis

## Abstract

Elevated levels of the metabolic by-products, including acidosis (i.e., high [H^+^]) and phosphate (P_i_) are putative agents of muscle fatigue; however, the mechanism through which they affect myosin’s function remain unclear. To elucidate these mechanisms, we directly examined the effect of acidosis (pH 6.5 vs. 7.4), alone and in combination with elevated levels of P_i_ on the force-generating capacity of a mini-ensemble of myosin using a laser trap assay. Acidosis decreased myosin’s average force-generating capacity by 20% (*p* < 0.05). The reduction was due to both a decrease in the force generated during each actomyosin interaction, as well as an increase in the number of binding events generating negative forces. Adding P_i_ to the acidic condition resulted in a quantitatively similar decrease in force but was solely due to an elimination of all high force-generating events (>2 pN), resulting from an acceleration of the myosin’s rate of detachment from actin. Acidosis and P_i_ also had distinct effects on myosin’s steady state ATPase rate with acidosis slowing it by ∼90% (*p* > 0.05), while the addition of P_i_ under acidic conditions caused a significant recovery in the ATPase rate. These data suggest that these two fatigue agents have distinct effects on myosin’s cross-bridge cycle that may underlie the synergistic effect that they have muscle force. Thus these data provide novel molecular insight into the mechanisms underlying the depressive effects of P_i_ and H^+^ on muscle contraction during fatigue.

## Introduction

Muscle fatigue from intense contractile activity is due, in large part, to the accumulation metabolic by-products, primarily hydrogen ions (H^+^) and phosphate (P_i_), inhibiting myosin’s ability to generate force and motion (Allen et al., 2008; Fitts, 2008; Debold et al., 2016). However, the molecular basis of this effect is still unclear, in part because the effect of P_i_ and H^+^ myosin’s force and motion-generating capacity have not been directly observed.

During fatigue P_i_ levels can exceed 15 mM (Cady et al., 1989) and are thought to inhibit force by accelerating myosin’s detachment from actin (Pate and Cooke, 1988, 1989; Takagi et al., 2004). A widely accepted model posits that P_i_ rebinds actomyosin in the ADP-bound state (AM.ADP), reverses myosin’s powerstroke, and induces detachment, restoring myosin to the pre-powerstroke state (Pate and Cooke, 1988; Takagi et al., 2004). However, this model is based, primarily, on the effects of P_i_ single fiber contractile properties (Hibberd et al., 1985a,b; Pate and Cooke, 1988, 1989; Dantzig et al., 1992; Debold et al., 2004; Caremani et al., 2008), which represent the average behavior of billions of myosin molecules, making it difficult to determine how P_i_ affects a single actomyosin cross-bridge. Indeed, our recent findings demonstrating that P_i_ increases velocity at low pH in the motility assay challenge the notion of P_i_ binding to actomyosin reversing the powerstroke, as do findings demonstrating the strain dependence of the effects of P_i_ (Caremani et al., 2008, 2013, 2015, Linari et al., 2010). The P_i_-induced increase in velocity at low pH led us to propose that the rebinding of P_i_ to actomyosin does not reverse the powerstroke, but instead induces myosin’s detachment in a post-powerstroke state (Debold et al., 2011, 2013)

However, we have only examined the effect of P_i_ on force at neutral pH (7.4) and not in combination with acidosis, which occurs in muscle fatigue (Cady et al., 1989). Under acidic conditions P_i_ is still thought to rebind to the AM.ADP state and induce detachment, but acidosis is thought to prolong the AM.ADP lifetime (Debold et al., 2008) increasing the vulnerability to P_i_ rebinding, and thus a potential mechanism for the synergistic effect these ions have on the force-generating capacity of muscle (Nosek et al., 1987; Nelson et al., 2014). However, this phenomenon has not been directly examined at the molecular level.

The molecular mechanisms underlying the depressive effects of acidosis (i.e., low pH) are also unclear, in part, because its role in the reduction of force remains highly controversial (Fitts, 2016; Westerblad, 2016), with some suggesting that it prevents rather than causes the loss of force during fatigue (Pedersen et al., 2004). Early work in muscle fibers suggested that a fatiguing level of acidosis depressed force by ∼50% and unloaded shortening velocity by ∼30% (Chase and Kushmerick, 1988; Cooke et al., 1988). But these observations were made well below mammalian physiological temperatures and subsequent observations at 30°C revealed that acidosis reduced force by only ∼10–20% (Pate et al., 1995; Westerblad et al., 1997; Knuth et al., 2006), leading some authors to conclude that acidosis plays little or no role in fatigue. However, the effect on unloaded shortening velocity appears to be much less temperature-sensitive, with a decrease from pH 7.0 to 6.2 causing reductions of 20–30% at both 15 and 30°C (Knuth et al., 2006). Indeed, the decrease in velocity leads to a 35–40% reduction in peak fiber power; a measure which is more relevant for understanding muscle fatigue (Knuth et al., 2006), but the molecular basis of these effects have not been elucidated.

## Methods

### Proteins

Fast skeletal muscle myosin (Margossian and Lowey, 1982; Debold et al., 2011) and actin were purified from (Pardee and Spudich, 1982) chicken pectoralis muscle as previously described. For the mini-ensemble laser trap assay, actin was labeled with 50% TRITC/phalliodin and 50% biotin/phalliodin. Animal tissue was obtained in accordance with the policies of the National Institutes of Health using a protocol approved by the Institutional Animal Care and Use Committee at the University of Massachusetts.

### Mini-Ensemble Laser Trap Assay

Myosin was loaded into a nitrocellulose coated flow-cell at 25 μg ml^-1^ in a high salt buffer (300 mM KCl, 25 mM imidazole, 1 mM EGTA, 4 mM MgCl_2_, and 1 mM dithiothreitol). The trapping buffer included fluorescently labeled actin and silica beads in a low salt buffer (60 mM KCl, 25 mM imidazole, 1 mM EGTA, and 4 mM MgCl_2_) with 100 μM MgATP, at pH 7.4 or 6.5 and either 0 or 15 mM added P_i_. Total ionic strength was kept constant 90 mM by varying KCl. Two 1 μm neutravidin-coated silica beads were trapped in time-shared optical traps and subsequently attached to a single actin filament (Debold et al., 2013). Once attached to actin, the trapped beads were separated to add 3–4 pN pretension to the filament. The trap stiffness was ∼0.02 pN/nm, which combined with the measures of displacement determined the force generated with each actomyosin interaction (see Supplementary Materials and Debold et al., 2013). At the myosin concentration used, the geometry of the assay indicates that ∼10, randomly oriented, myosin molecules were available to interact with the single actin filament (Debold et al., 2013).

### ATPase Assay

The effect of acidosis and P_i_ on myosin’s steady state rate ATP hydrolysis was determined using an NADH-linked assay solution with the heavy meromyosin (HMM) fragment of whole myosin at 30°C, using established methods (De La Cruz et al., 2000). HMM was dialyzed into the appropriate low salt buffer under control conditions (20 mM KCl, 25 mM imidazole, 1 mM EGTA, 4 mM MgCl_2_, and 1 mM dithiothreitol at pH 7.4). 10 mM P_i_ was used instead of the 15 mM used in the laser trap assay in order to keep the ionic strength sufficiently low for the ATP hydrolysis rate to approach saturation at the actin concentrations used (0–50 μM). Previous evidence demonstrates that P_i_ exerts ∼70% of its effect on muscle force between 0 and 10 mM, with only minor additional decrement (<5%) caused by 15 mM (Tesi et al., 2000), thus the differences between the two different levels of P_i_ used are likely minimal.

## Results

**FIGURE 1:**
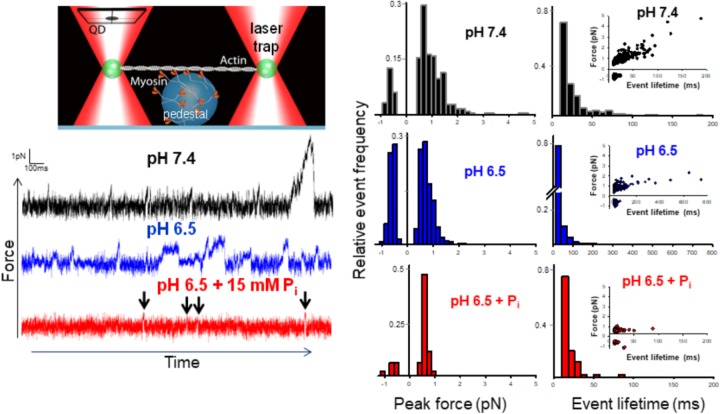
Effect of acidosis and phosphate on myosin’s force-generating capacity. Schematic of the mini-ensemble of myosin molecules in a laser trap assay (on large sphere), showing a single actin filament pulled taut between two optically trapped 1 um silica beads, top left. Raw displacement vs. time records with actomyosin binding events appearing as reductions in the variance and displacements from the mean signal under each condition indicated (see section “Methods”). Determination of the stiffness of the laser trap (0.02 pN/nm) enabled converting these displacements into forces. An automated routine written MatLab was used to determine the peak force for each event (see section “Methods” and Supplementary Materials). Scored events were used to construct histograms and plotted as a function of the total number of events in a condition (middle set of panels). Then median forces were 0.78 ± 0.27 (pH 7.4, black), 0.63 ± 0.13 (pH 6.5, blue)^∗^, and 0.59 ± 0.06 pN (pH 6.5 + 15 mM P_i_, red)^∗^ for control, acidosis, and acidosis with added P_i_ respectively, ^∗^indicates significantly (*p* < 0.05) lower than control. The lifetime of each event was also determined using the same automated routine (see section “Methods” and Supplementary Materials) and histograms constructed for each condition (right panels). Insets show peak force plotted as a function of event lifetime. Median event lifetimes were 18 ± 1, 15 ± 1^∗^, and 15 ± 0.5^∗^ ms for control (black), acidosis (blue), and acidosis + P_i_ respectively (red) (^∗^indicates significantly different from control, *p* < 0.05). The data shown represent between 329, 623, and 87 binding events for control, acidosis, and acidosis + P_i_ respectively.

The mini-ensemble of myosin molecules stochastically interacted with the actin filament to produce a range of low and high force-generating events (**Figure 1**). We identified the peak force and lifetime of these events with a customized event-detection algorithm (see Supplementary Materials and Longyear et al., 2017). Decreasing the pH from 7.4 to 6.5 significantly (*p* < 0.05) decreased the average peak force by ∼20% in the mini-ensemble laser trap assay. Examination of the distribution of events revealed this was caused by a decrease in the frequency of high force-generating events (events > 2 pN), as well as an increase in the frequency of negative force-generating events (events occurring opposite of the predominate direction). The addition of P_i_ at pH 6.5 caused a similar depression in force, but was largely due to the elimination of higher force-generating events, accompanied by a decrease in the frequency of negative force events.

Event lifetimes appeared exponentially distributed (**Figure 1**), and in the case of the control were linearly related to the forces generated (**Figure 1**, far right panels). Decreasing the pH to 6.5 caused a reduction in the number of high force-generating events, but also caused the slope of the force vs. duration to decrease significantly (0.0273 ± 0.0015 vs. 0.0044 ± 0.0005, *p* < 0.05). This finding indicates that the binding events lasted longer at pH 6.5 despite generating less force. This suggests that acidosis slows the rate of detachment for the longer duration events. By contrast, the addition of P_i_ at pH 6.5 caused a pronounced reduction in longer event lifetimes with all but two binding events lasting for <50 ms, suggesting an accelerated rate of detachment.

**FIGURE 2:**
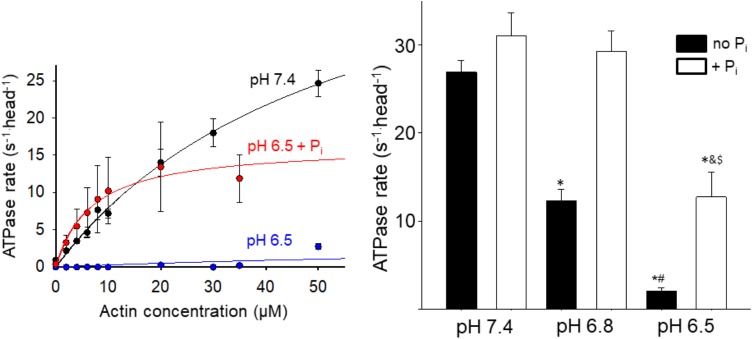
Effect of acidosis and phosphate on steady state ATPase. The rate of product release from HMM as a function of actin concentration was determined using an NADH-linked assay (left graph, see Supplementary Materials for details), under control (pH 7.4 no added P_i_), acidic (pH 6.5), and acidic plus P_i_ (pH 6.5 + 10 mM P_i_) conditions. Each point represents the mean ± SEM between 7 and 33 measures at each actin concentration. The maximum rates obtained under each condition (including pH 6.8 and pH 6.8 + P_i_) are plotted in the graph on the right side. Bars represent mean ± SEM for the concentration of actin yielding the highest rate. ^∗^Indicates significantly (*p* < 0.05) different from control, ^#^indicates significantly different from pH 6.8 no P_i_, ^&^indicates significantly different from pH 6.5 no P_i_, and ^$^indicates significantly different from pH 6.8 + P_i_.

To gain insight into the effect acidosis and P_i_ might have on steps of the cross-bridge cycle occurring off actin we measured the effect on the steady state ATPase rate in solution (**Figure 2**). Acidosis strongly depressed actin-activated V_max_, causing a 60% reduction at pH 6.8 and ∼90% reduction at pH 6.5 (**Figure 2**). In contrast, the addition of 10 mM P_i_ at low pH significantly increased myosin’s ATPase rate. The effect was small at pH 7.4 but became more pronounced as the pH was decreased (**Figure 2**). Despite the recovery it should be noted that at pH 6.5 in the presence of P_i_ the ATPase remained significantly slower than the control at pH 7.4.

## Discussion

Both acidosis alone and in combination with P_i_ significantly reduced myosin’s force-generating capacity; however, the mechanism underlying this effect appears to be driven by distinct mechanisms. Acidosis decreased force by decreasing the number of high force-generating events and increasing the frequency of negative forces, while the addition of P_i_ reduced force by eliminating all high force/long duration binding events (**Figure 1**).

The decrease in high force-generating events under acidic conditions suggests that less myosin heads were strongly bound during a given interaction, indicative of slowing myosin’s weak-to-strong binding transition. This suggests that one or more of the steps of myosin’s cross-bridge cycle that occur off actin are slowed by acidosis. One possibility is that myosin’s putative rate-limiting step, P_i_-release (Lymn and Taylor, 1971), which occurs closely or concomitantly with strong-binding (Takagi et al., 2004), is slowed by acidosis. This possibility is consistent with the acidosis-induced reduction of the myosin steady state ATPase rate (**Figure 2**).

The increase in negative force events under acidic conditions is consistent with prior observations demonstrating that acidosis increases the frequency of non-productive single actomyosin interactions (Debold et al., 2008). Because beads in a laser trap experience Brownian motion, non-productive actomyosin interactions cause the frequency of negative displacement binding events to increase (Debold et al., 2008). Myosin’s powerstroke is thought to be re-primed with the hydrolysis of ATP, off actin (Steffen et al., 2003), therefore an increase in non-productive interactions may indicate that acidosis slows the rate of ATP hydrolysis by myosin.

The decrease in the slope of the force vs. event lifetime relationship with acidosis (**Figure 1**) indicates that at any given force, actomyosin binding events lasted longer than at pH 7.4. This observation is consistent with acidosis prolonging single actomyosin interactions, which has been attributed to a slowing rate of ADP-release (Debold et al., 2008, 2012). This mechanism, therefore, provides a molecular mechanism for the acidosis-induced decrease in unloaded shortening velocity observed in the motility assay (Debold et al., 2008, 2011), and in skinned muscle fibers (Knuth et al., 2006).

In contrast to the effects of acidosis alone, increasing P_i_ at low pH reduced force solely by reducing the event lifetime (**Figure 1**). Indeed, the combined effects of acidosis and P_i_ on force are nearly identical to the effect of P_i_ alone (Debold et al., 2013), where virtually all of the high force/long duration events are eliminated by elevated P_i_. This suggests that any effect of acidosis to prolong the actomyosin interaction is opposed by the P_i_-induced detachment from an AM.ADP state (Hibberd et al., 1985b; Webb et al., 1986). Indeed, the prolongation of the AM.ADP state by acidosis may extend the lifetime of the state to which P_i_ rebinds to induce detachment. Thus, acidosis may make actomyosin more vulnerable to the rebinding of P_i_, which may provide a molecular basis for the synergistic effects P_i_ and H^+^ have on muscle force (Nelson et al., 2014).

Another key question is what happens to myosin following this P_i_-induced detachment from actin? The conventional view posits that the rebinding of P_i_ to AM.ADP induces a reversal of the powerstroke before myosin detaches from actin; thus myosin is restored to a pre-powerstroke AM.ADP P_i_ state (Pate and Cooke, 1988; Takagi et al., 2004). However, this view fails to account for the P_i_-induced increase in actin filament velocity at low pH which we observed in the motility assay (Debold et al., 2011). Therefore, we proposed an alternative model in which P_i_ induces detachment from a post-powerstroke state through a branch in the normal cross-bridge cycle (Debold et al., 2011, 2013). This model is able to accurately reproduce the P_i_-induced increase in velocity at low pH (Debold et al., 2011), suggesting that myosin detaches from actin in a post-powerstroke state, and completes it ATPase off of actin. Indeed, the effect of acidosis and P_i_ on myosin’s ATPase rate (**Figure 2**) is strikingly similar to the effect on velocity providing further support for our model (Debold et al., 2011, 2012). However, this similarity may be coincidental as velocity, *in situ*, is thought to be limited by the ADP-release (Nyitrai et al., 2006), while the ATPase rate, in solution, is believed to be limited by P_i_-release (Lymn and Taylor, 1971); rates which are at least an order of magnitude different. In our model, P_i_ increases velocity at low pH by accelerating detachment from a post-powerstroke state (Debold et al., 2011). The ATPase data in the present study suggest that, under acidic conditions, once myosin is detached from actin by P_i_ it completes product release faster off actin than it does when strongly bound to actin (**Figure 2**). Thus acidosis may have a greater effect on ADP-release when myosin is strongly bound to actin than once it has been detached by P_i_.

## Conclusion

Elevated levels of the fatigue agents, P_i_ and H^+^, directly inhibit myosin’s force-generating capacity; however, this seems to arise from distinctly different molecular mechanisms. Acidosis appears to slow both myosin’s weak-to-strong binding transition and its rate of detachment from actin, while P_i_ reduces force by accelerating detachment from actin. The P_i_-induced acceleration in detachment may mitigate some of the effects of acidosis on velocity and the hydrolysis rate, but this likely comes at the expense of force generation. Thus these data may provide a molecular basis for the putative synergistic effects of P_i_ and H^+^ on muscle force in muscle fibers (Nosek et al., 1987; Nelson et al., 2014) and during fatigue (Cady et al., 1989).

## Author Contributions

ED conceived the idea, collected and analyzed the data, and wrote the manuscript. MW collected data and helped to analyze the data and was involved in the preparation of the manuscript.

## Conflict of Interest Statement

The authors declare that the research was conducted in the absence of any commercial or financial relationships that could be construed as a potential conflict of interest.
