# Supplementary material for: Acidosis and Phosphate Directly Reduce Myosin’s Force-Generating Capacity Through Distinct Molecular Mechanisms
Source: Front Physiol. 2018 Jul 10;9:862. doi: 10.3389/fphys.2018.00862 (PMC6048269; doi:10.3389/fphys.2018.00862)
Supplement: Supplementary file 1 [file Image_1.PDF]

Supplemental Materials

ATPase assay

The rate NADH oxidation was monitored over several minutes by measuring the absorbance changes at 340 nm, based on NADH an extinction coefficient of ϵ_{340} = 6,220 $M^{-1} \cdot cm^{-1}$ using a spectrophotometer. The rate of NADH oxidation was plotted and fit to a lit with the slope taken as the stead-state value and subsequently converted to Pi/sec/myosin head based on the HMM concentration in the assay. These measurements were obtained over a range of actin concentrations (1-50uM) and used to obtain values of V_{max} and K_m. To compare among conditions the ATPase rate at saturating actin concentrations were used in the ANOVA.

Analysis of mini-ensemble laser trap assay data

These data were analyzed using a custom Matlab program previously described (Longyear et al. 2017). Briefly, the program employs a threshold algorithm that scores displacements had greater than 8 nm from a defined baseline that have a duration ≥ 10 ms. These values were chosen based on the lower limit of the average displacement for two-headed skeletal muscle myosin (Tyska and Warshaw 1999) and the expected lifetime of actomyosin strong-binding at 100uM ATP 10 ms (White and Taylor 1976). The highest force sustained for ≥ 10 ms during a binding event was taken as the peak force. Event duration was determined based on the time spent > 8nm from baseline and event frequency was determined by dividing the number of events by the total time of the displacement recording (typically 10-30sec each).

Statistical Analysis

Differences among the different conditions in the laser trap data were determined using a non-parametric Kruskal-Wallis ANOVA and associated post hoc test was used to locate differences. Non-parametric tests were used because the data force and lifetime data were not Normally-distributed (Shapiro-Wilk test) and had unequal variances (Unequal Variance test).

Differences among the difference conditions of the ATPase assay were determined using a one-way ANOVA followed by a Tukey's HSD to locate the differences. The ATPase data were fit with the Michealis-Menten relation to derive values for V_{max} and K_m. All statistical analyses were performed using SigmaPlot® 11.0.
